# Supplementary figures and images for: Structural characterization of ligand binding and pH-specific enzymatic activity of mouse Acidic Mammalian Chitinase
Source: bioRxiv. 2024 Mar 25:2023.06.03.542675. Preprint. [Version 3] doi: 10.1101/2023.06.03.542675 (PMC10312649; doi:10.1101/2023.06.03.542675)

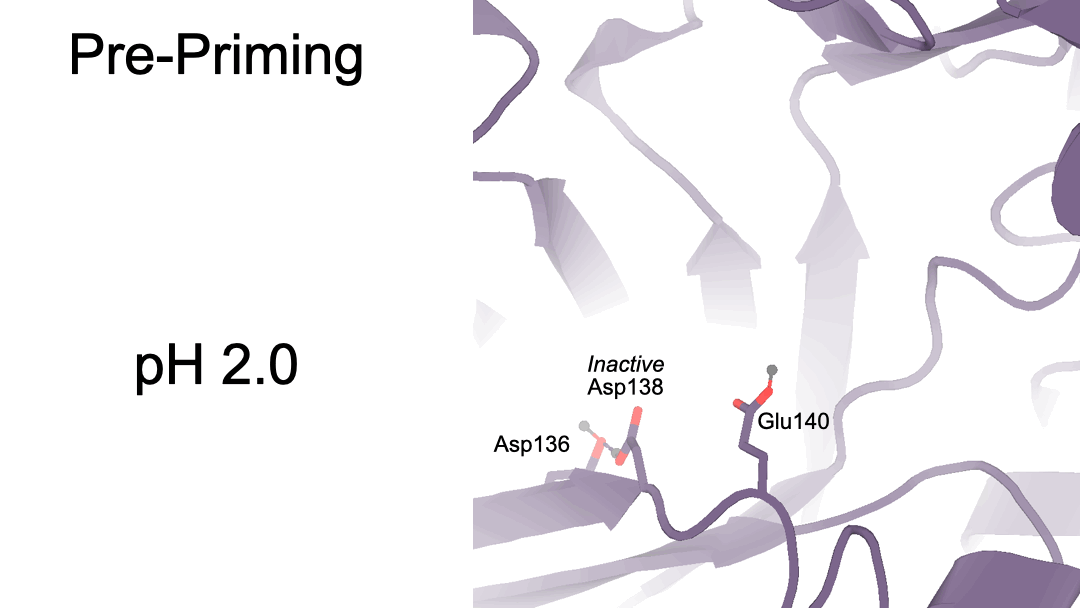

Supplement: Supplement 3 [file media-3.gif]

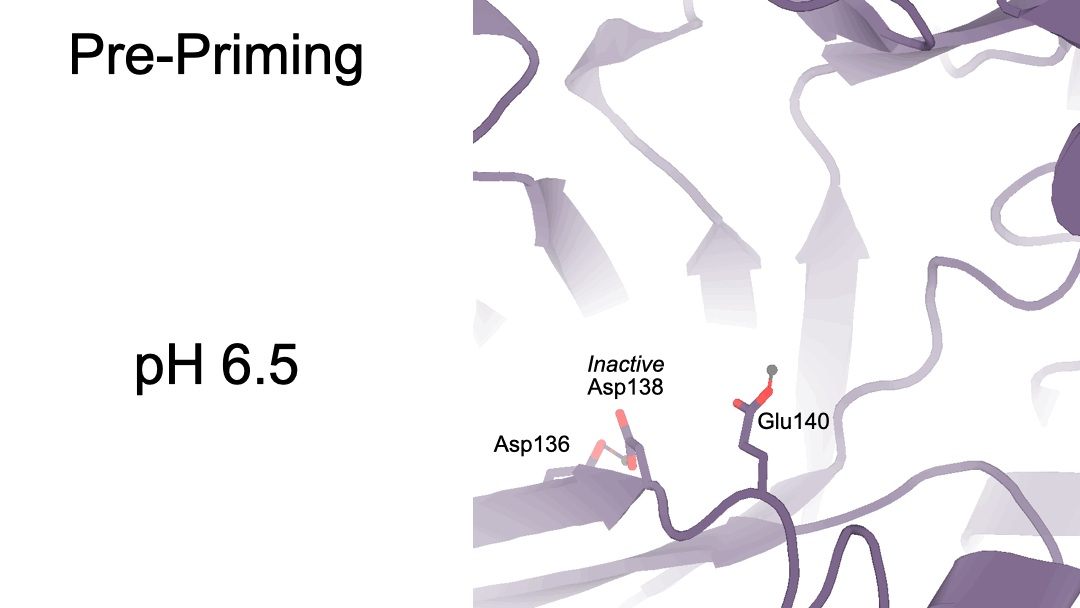

Supplement: Supplement 4 [file media-4.gif]
