# Supplementary material for: Structural characterization of ligand binding and pH-specific enzymatic activity of mouse Acidic Mammalian Chitinase
Source: bioRxiv. 2023 Jun 28:2023.06.03.542675. Preprint. [Version 2] doi: 10.1101/2023.06.03.542675 (PMC10312649; doi:10.1101/2023.06.03.542675)
Supplement: Supplementary file 1 [file NIHPP2023.06.03.542675v2-supplement-5.pdf]

bioRxiv preprint doi: https://doi.org/10.1101/2023.06.03.543575; this version posted June 3, 2023. The copyright holder for this preprint (which was not certified by peer review) is the author/funder, who has granted bioRxiv a license to display the preprint in perpetuity. It is made available under aCC-BY 4.0 International license.

Supplemental Figures

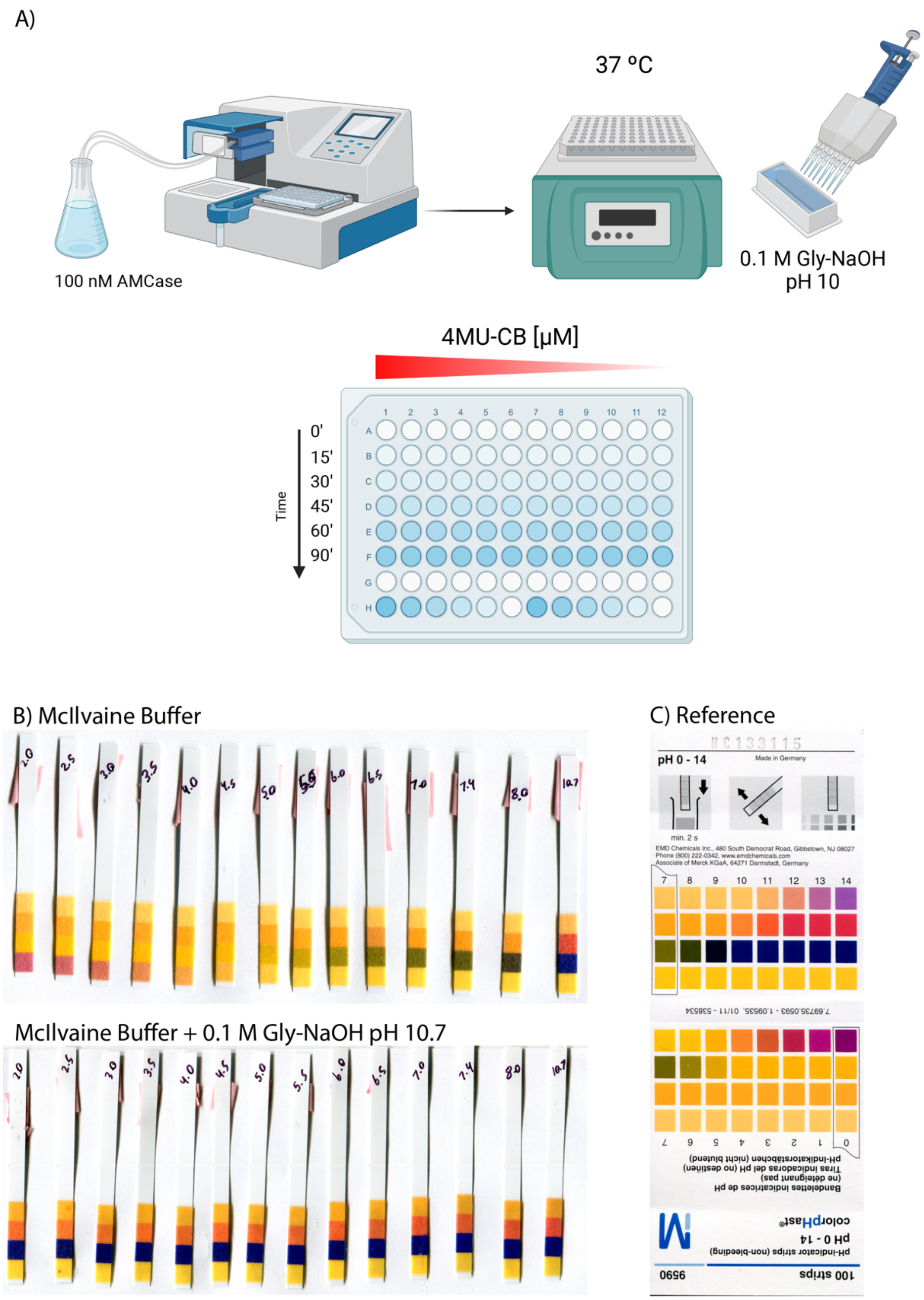

Supplemental Figure 1 | pH of reaction solution before and after quenching with 0.1 M Gly-NaOH pH 10.7

A) Schematic of modified endpoint 4MU-chitobioside assay. **B)** Reaction pH before and after quenching with 0.1 M Gly-NaOH pH 10.7, and **C)** a pH strip reference sheet.

bioRxiv preprint doi: [illegible]; this version posted June 3, 2023. The copyright holder for this preprint (which was not certified by peer review) is the author/funder, who has granted bioRxiv a license to display the preprint in perpetuity. It is made available under aCC-BY 4.0 International license.

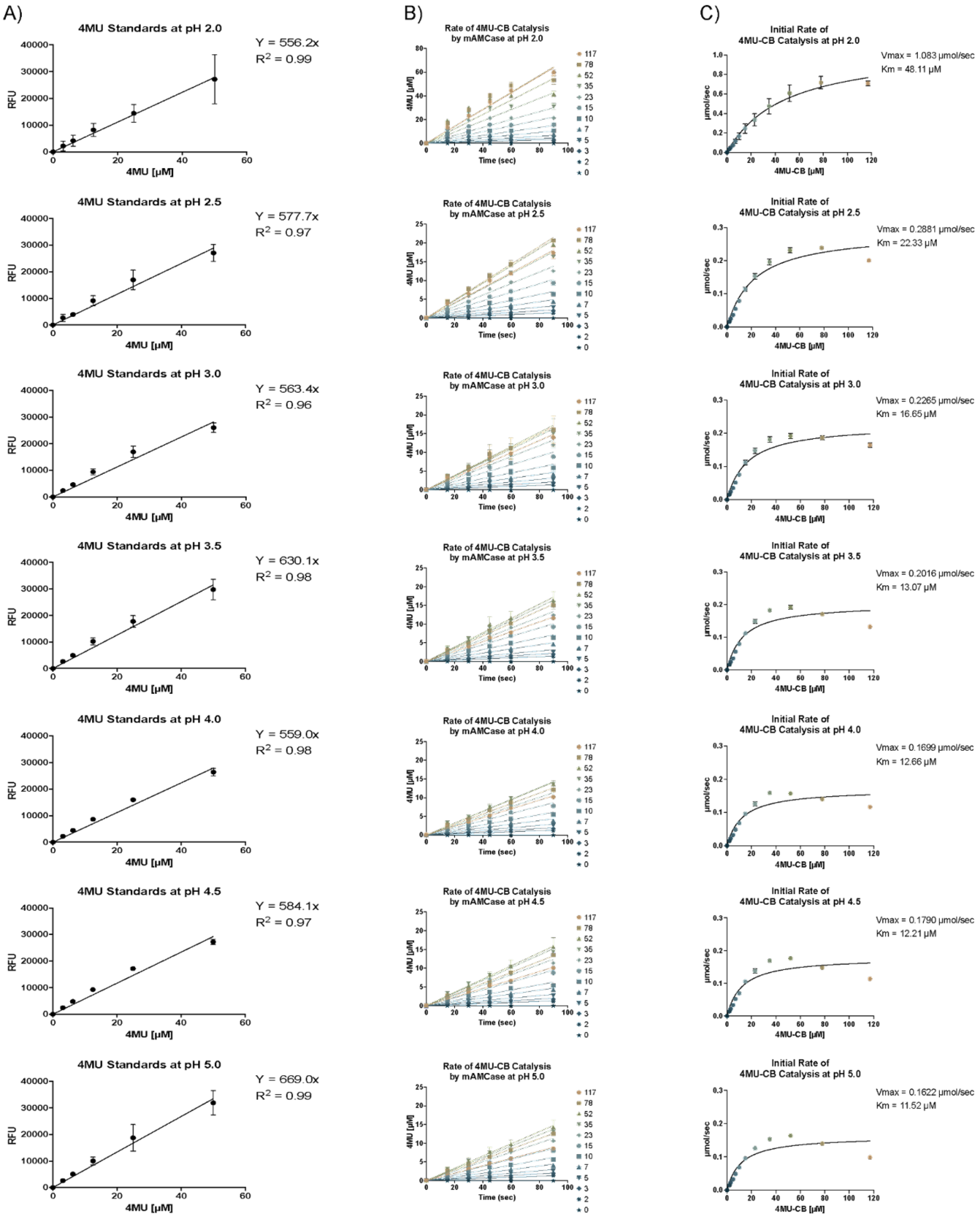

bioRxiv preprint doi: https://doi.org/10.1101/2023.06.03.543575; this version posted June 3, 2023. The copyright holder for this preprint (which was not certified by peer review) is the author/funder, who has granted bioRxiv a license to display the preprint in perpetuity. It is made available under aCC-BY 4.0 International license.

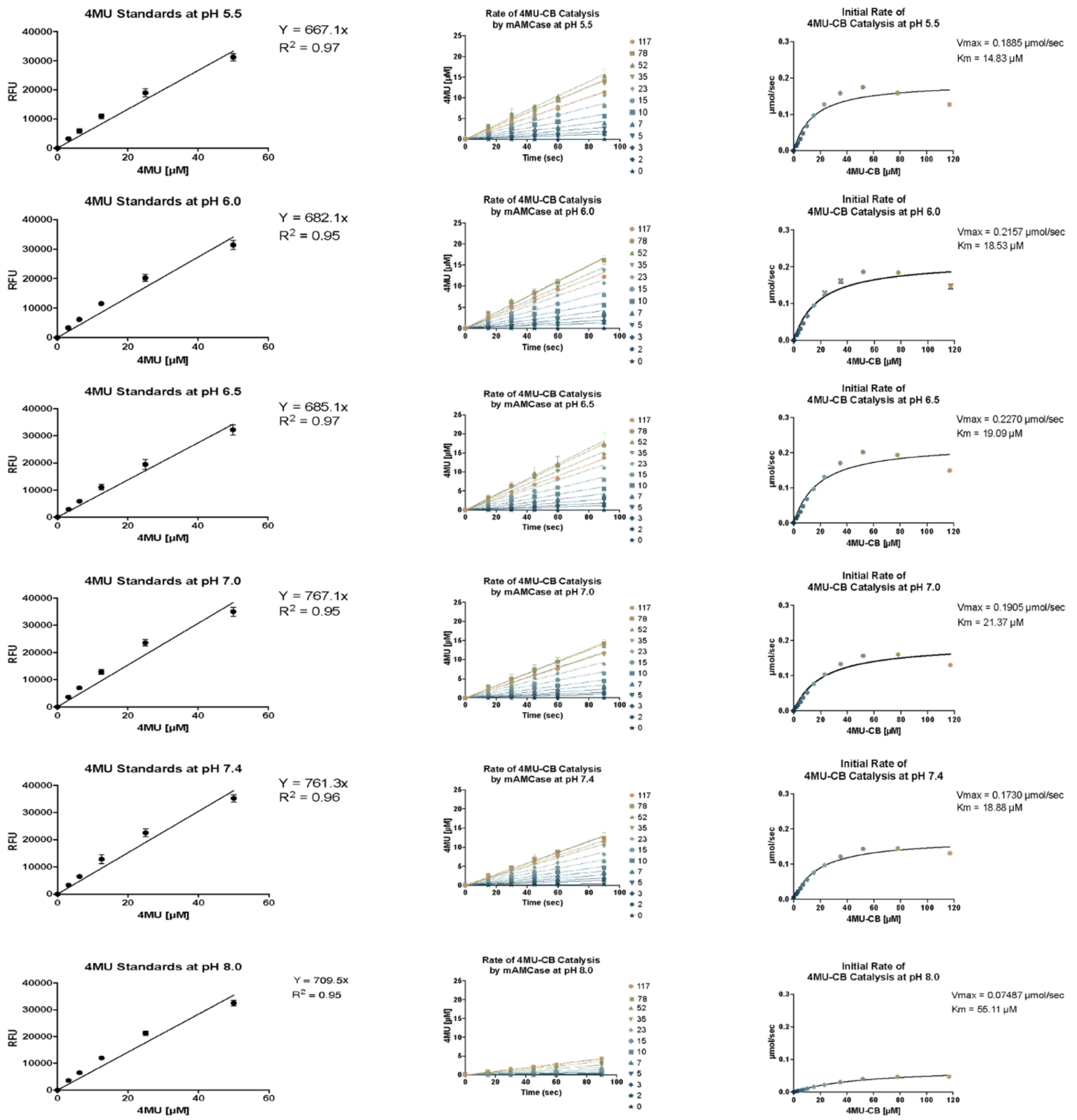

Supplemental Figure 2 | Kinetics of 4MU-chitobioside catalysis by mAMCase catalytic domain at various pH.

A) A linear fit forced through Y = 0 is used to generate the standard curve for converting RFU to 4MU [µM]. Each data point represents n = 8 with error bars representing the standard deviation. **B)** 4MU fluorescence (RFU) is plotted as a function of time (sec). Each data point represents n = 4 with error bars representing the standard deviation. A linear fit is applied to each concentration of 4MU-chitobioside to calculate an initial rate. RFU is converted to µM using a 4MU standard curve. **C)** The rate of 4MU-chitobioside catalysis (1/sec) by mAMCase catalytic domain is plotted as a function of 4MU-chitobioside concentration (µM). Each data point represents n = 4 with error bars representing the standard deviation. Michaelis-Menten equation without substrate inhibition was used to estimate the k_{cat} and K_M from the initial rate of reaction at various substrate concentrations.

bioRxiv preprint doi: https://doi.org/10.1101/[illegible]; this version posted June [illegible], 2023. The copyright holder for this preprint (which was not certified by peer review) is the author/funder, who has granted bioRxiv a license to display the preprint in perpetuity. It is made available under aCC-BY 4.0 International license.

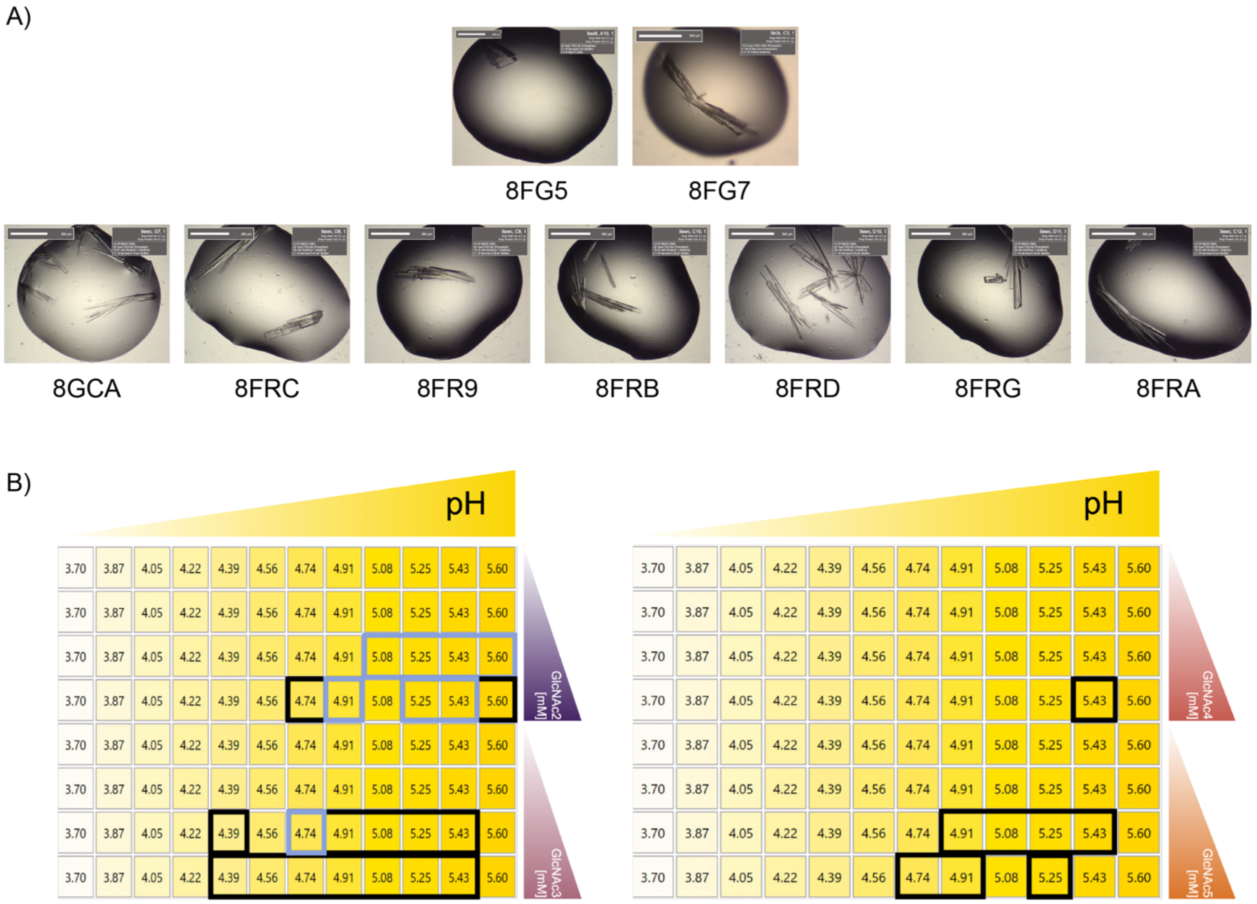

Supplemental Figure 3 | 96-well plate layout of crystallization conditions.
A) Brightfield view of crystals used to determine the structures reported in this paper. **B)** Hanging drop crystallization trays were set up as a 2-condition gradient to identify optimal crystallization conditions for AMCase + $GlcNAc_n$. pH increased along the X-axis from pH 3.70 to 5.60. Ligand concentration increased along the Y-axis from 0 mM to 29 mM [$GlcNAc_2$], 19 mM [$GlcNAc_3$], 10 mM [$GlcNAc_4$], or 8 mM [$GlcNAc_5$]. Black boxes indicate conditions where crystals grew. Lilac boxes indicate conditions for structures reported in this paper.

bioRxiv preprint doi: https://doi.org/10.1101/2023.06.03.[illegible]; this version posted June [illegible], 2023. The copyright holder for this preprint (which was not certified by peer review) is the author/funder, who has granted bioRxiv a license to display the preprint in perpetuity. It is made available under aCC-BY 4.0 International license.

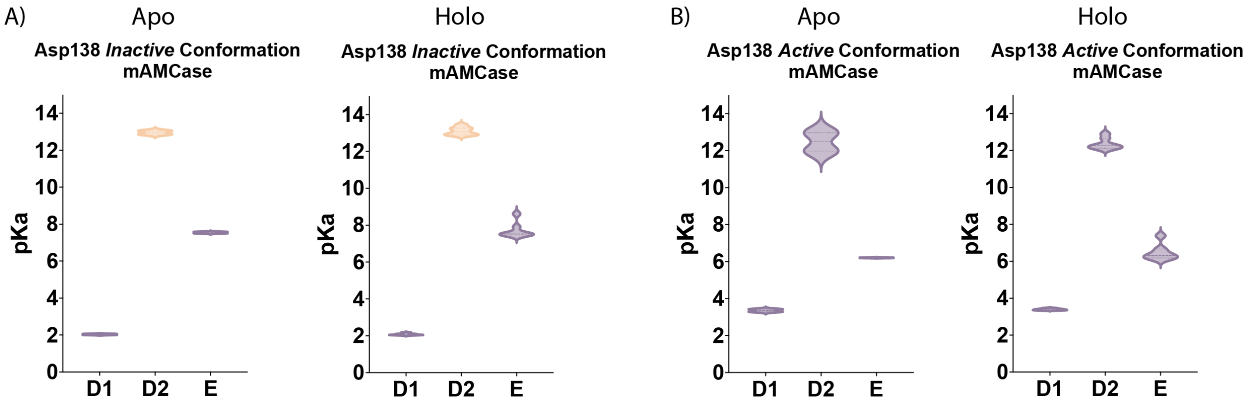

Supplemental Figure 4 | pKa of apo and holo mAMCase in the D2 *inactive* and *active* conformation.

PDB ID: 8FG5, 8FG7 (*apo*); 8GCA, 8FRC, 8FR9, 8FRB, 8FRD, 8FRG, 8FRA (*holo*). Distribution of pKa across Asp136, Asp138, Glu140 between **A)** apo and **B)** holo mAMCase structures in the *inactive* or *active* conformation.

bioRxiv preprint doi: https://doi.org/10.1101/[illegible]; this version posted [illegible], 2023. The copyright holder for this preprint (which was not certified by peer review) is the author/funder, who has granted bioRxiv a license to display the preprint in perpetuity. It is made available under aCC-BY 4.0 International license.

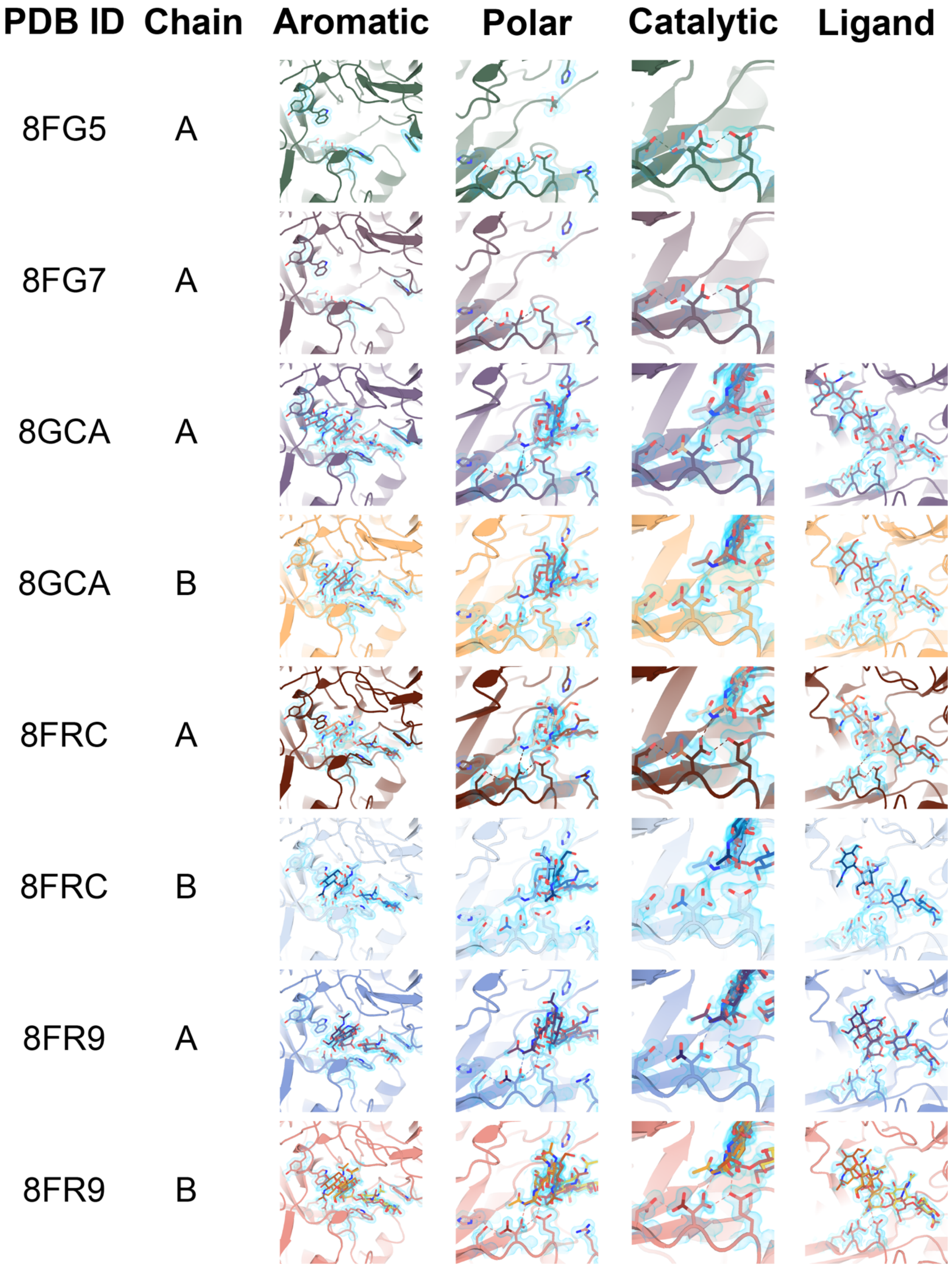

bioRxiv preprint doi: https://doi.org/10.1101/2023.06.03.543475; this version posted June 3, 2023. The copyright holder for this preprint (which was not certified by peer review) is the author/funder, who has granted bioRxiv a license to display the preprint in perpetuity. It is made available under aCC-BY 4.0 International license.

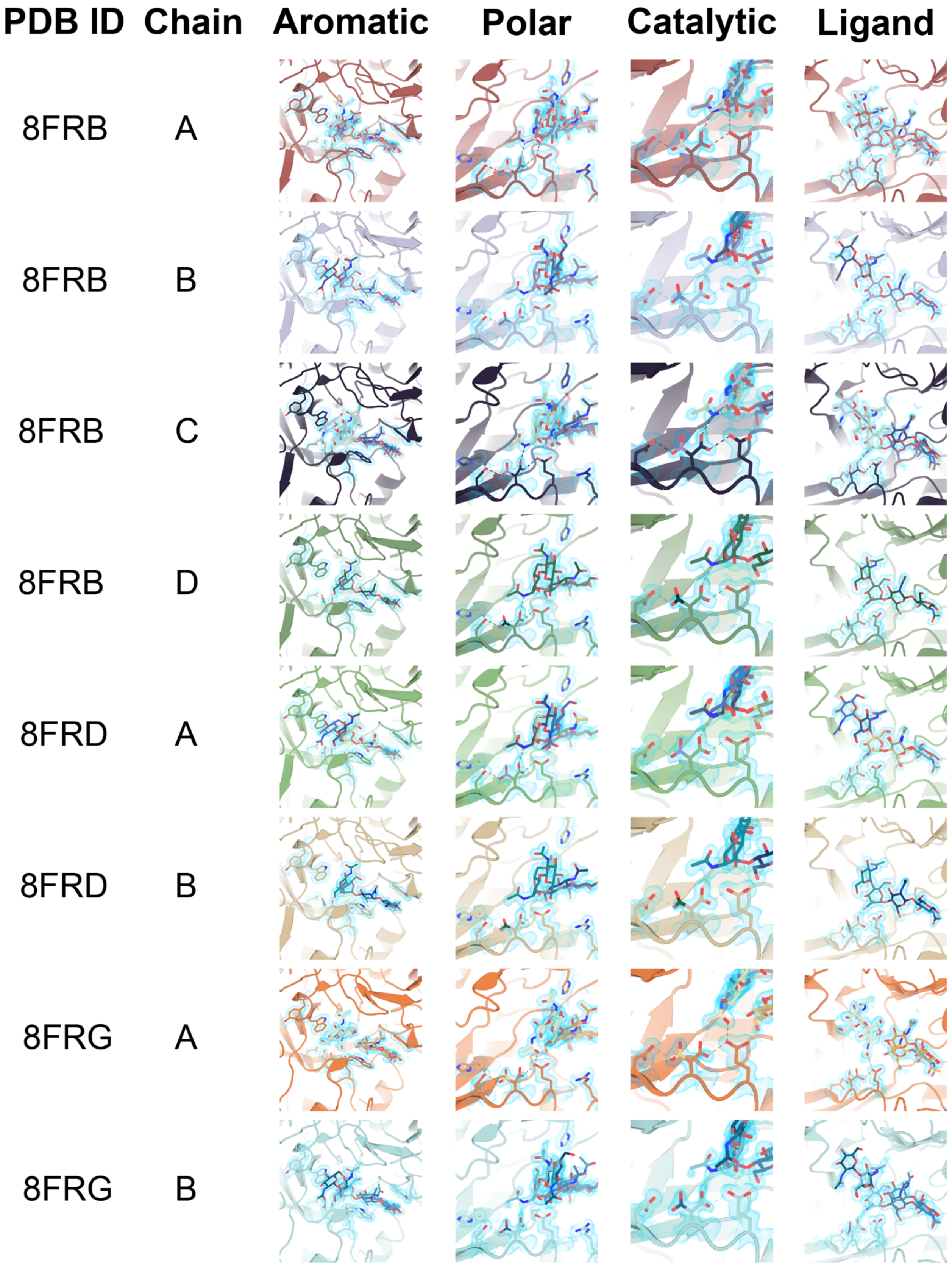

bioRxiv preprint doi: https://doi.org/10.1101/2023.06.03.[illegible]; this version posted June 3, 2023. The copyright holder for this preprint (which was not certified by peer review) is the author/funder, who has granted bioRxiv a license to display the preprint in perpetuity. It is made available under aCC-BY 4.0 International license.

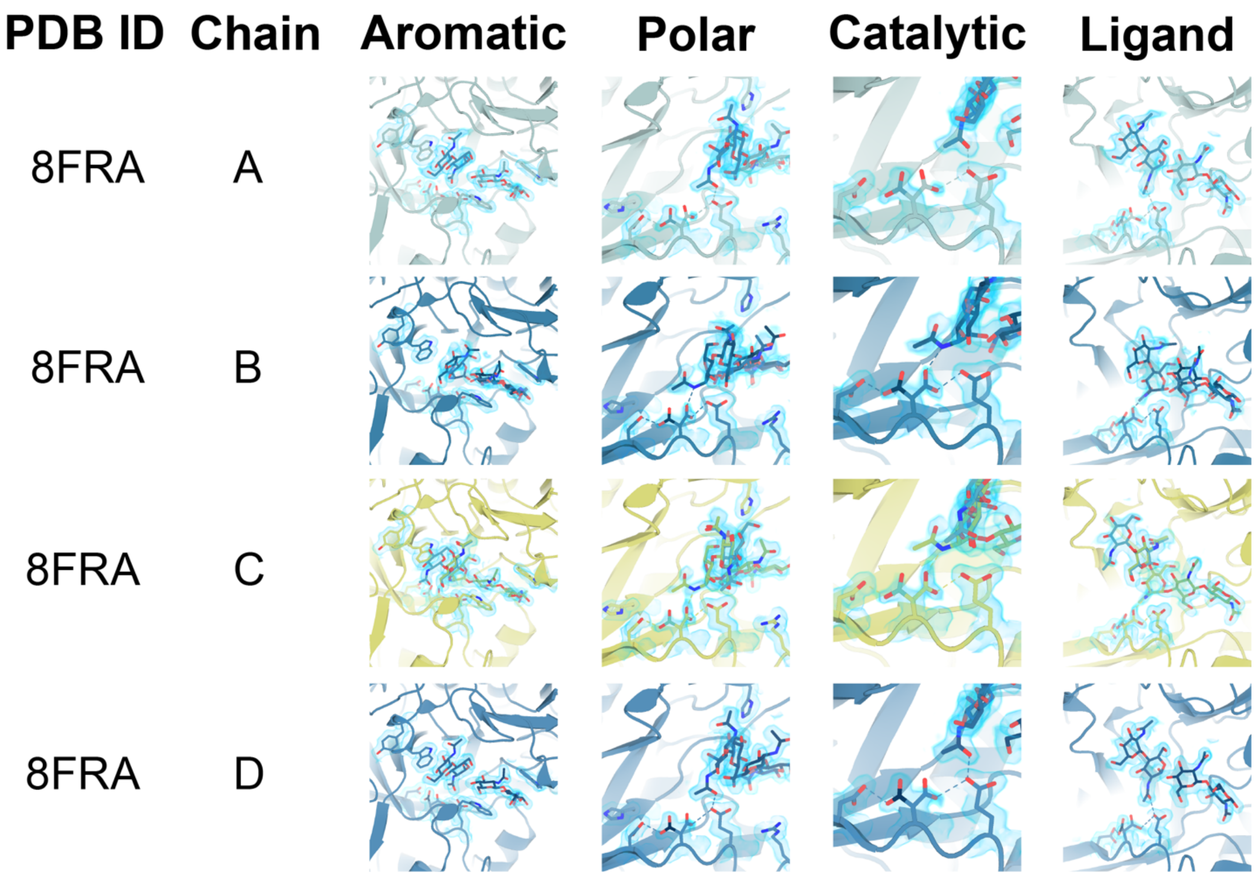

Supplemental Figure 5 | Overview of key residues for mAMCase activity.
A) Stick representation of ligand and aromatic residues Trp31, Tyr34, Trp99, and Trp218 in the active site with 2mFo-DFc map shown as a 1.2 Å contour (blue). **B)** Stick representation of ligand and polar residues Arg145, His208, Asp213, and His269 in the active site with 2mFo-DFc map shown as a 1.2 Å contour (blue). **C, D)** Stick representation of ligand and catalytic residues Asp136, Asp138, and Glu140 in the active site with 2mFo-DFc map shown as a 1.2 Å contour (blue).

bioRxiv preprint doi: https://doi.org/10.1101/2023.06.03.543575; this version posted June 28, 2023. The copyright holder for this preprint (which was not certified by peer review) is the author/funder, who has granted bioRxiv a license to display the preprint in perpetuity. It is made available under aCC-BY 4.0 International license.

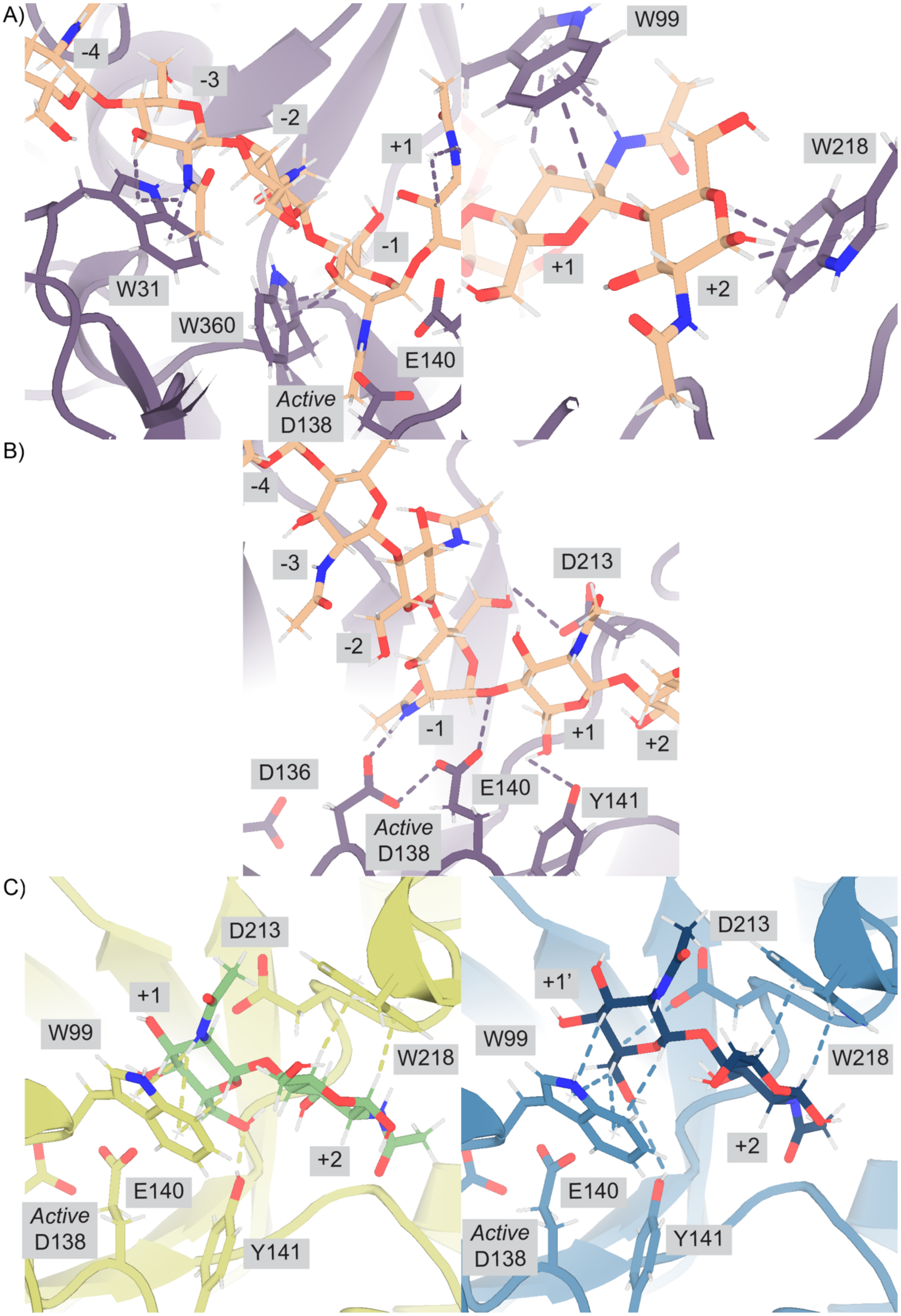

Supplemental Figure 6 | Protein-ligand interactions between mAMCase and chitin.
A) PDB ID: 8GCA, chain A with GlcNAc$_6$ modeled for viewing simplicity. Stick representation highlighting the stabilizing H-π interactions between Trp31, Trp360, and Trp218 and the -3, -1,

bioRxiv preprint doi: https://doi.org/10.1101/2023.06.03.543575; this version posted June 3, 2023. The copyright holder for this preprint (which was not certified by peer review) is the author/funder, who has granted bioRxiv a license to display the preprint in perpetuity. It is made available under aCC-BY 4.0 International license.

+1, and +2 sugars, respectively. **B)** PDB ID: 8GCA, chain A with $GlcNAc_6$ modeled for viewing simplicity. Stick representation highlighting the stabilizing hydrogen bond interactions between the -1 sugar and Asp138 (2.6 Å) and Asp213 (3.4 Å), and between the +1 sugar and Tyr141 (3.0 Å). Glu140 is 2.8 Å from the glycosidic oxygen bridging the -1 and +1 sugars. **C)** PDB ID: 8FRA, chains C (left) and D (right). Stick representation highlighting the stabilizing hydrogen bond interactions that we hypothesize stabilize the +1 sugar (left; chain A) and the +1' sugar-binding subsite (right; chain B).

bioRxiv preprint doi: https://doi.org/10.1101/[illegible]; this version posted June [illegible], 2023. The copyright holder for this preprint (which was not certified by peer review) is the author/funder, who has granted bioRxiv a license to display the preprint in perpetuity. It is made available under aCC-BY 4.0 International license.

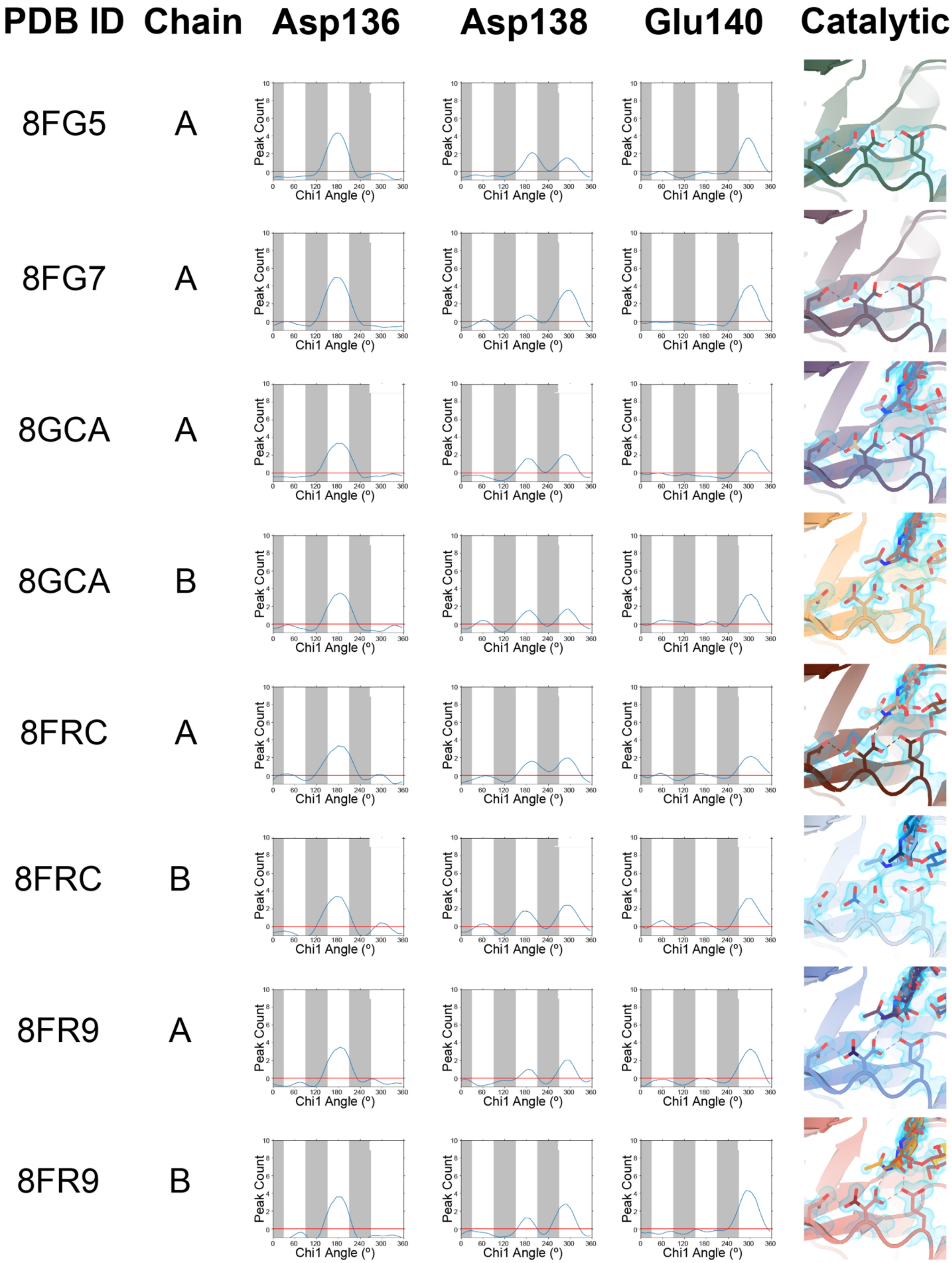

bioRxiv preprint doi: https://doi.org/10.1101/[illegible]; this version posted [illegible], 2023. The copyright holder for this preprint (which was not certified by peer review) is the author/funder, who has granted bioRxiv a license to display the preprint in perpetuity. It is made available under aCC-BY 4.0 International license.

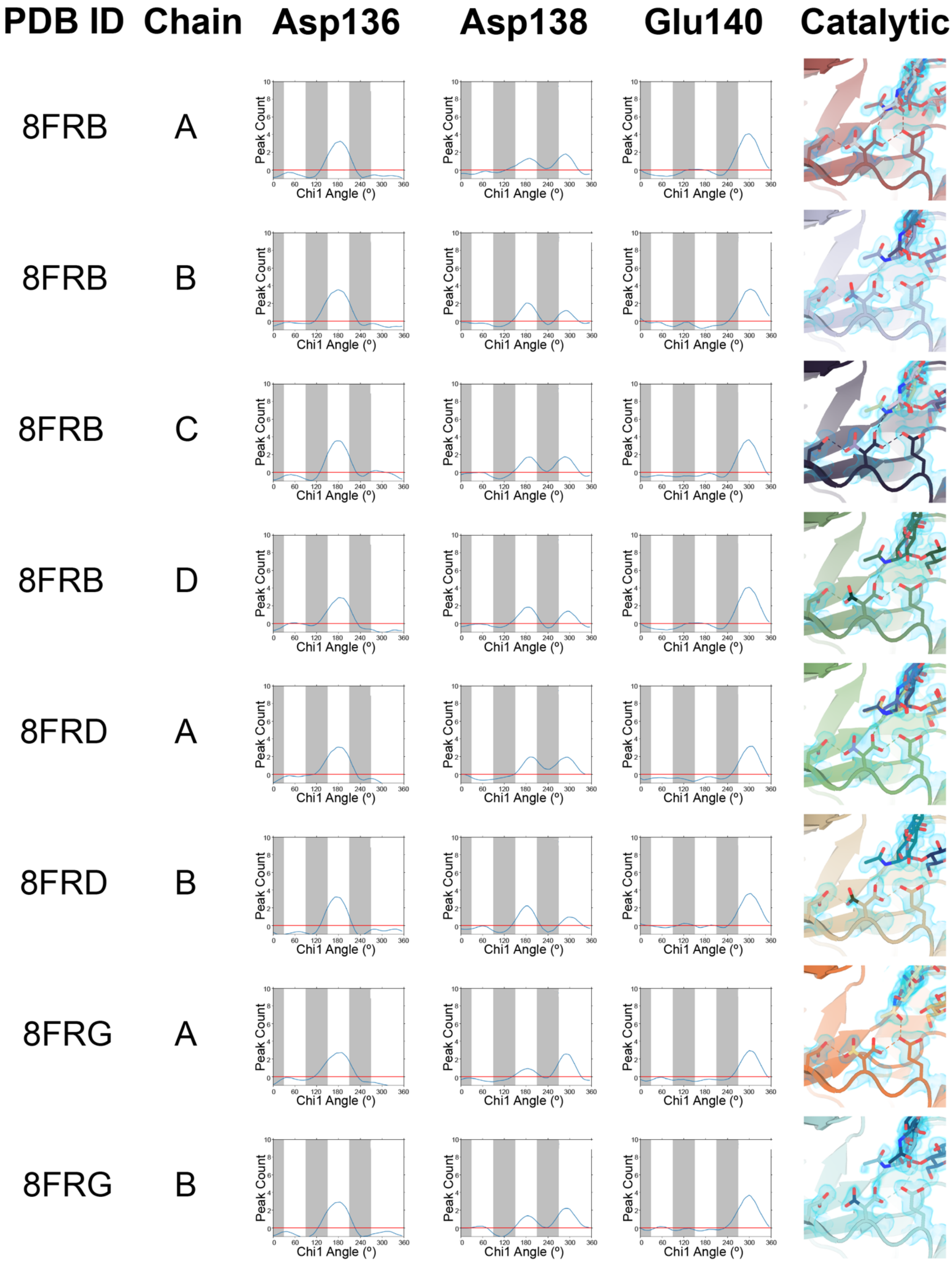

bioRxiv preprint doi: https://doi.org/10.1101/2023.06.03.543475; this version posted June 3, 2023. The copyright holder for this preprint (which was not certified by peer review) is the author/funder, who has granted bioRxiv a license to display the preprint in perpetuity. It is made available under aCC-BY 4.0 International license.

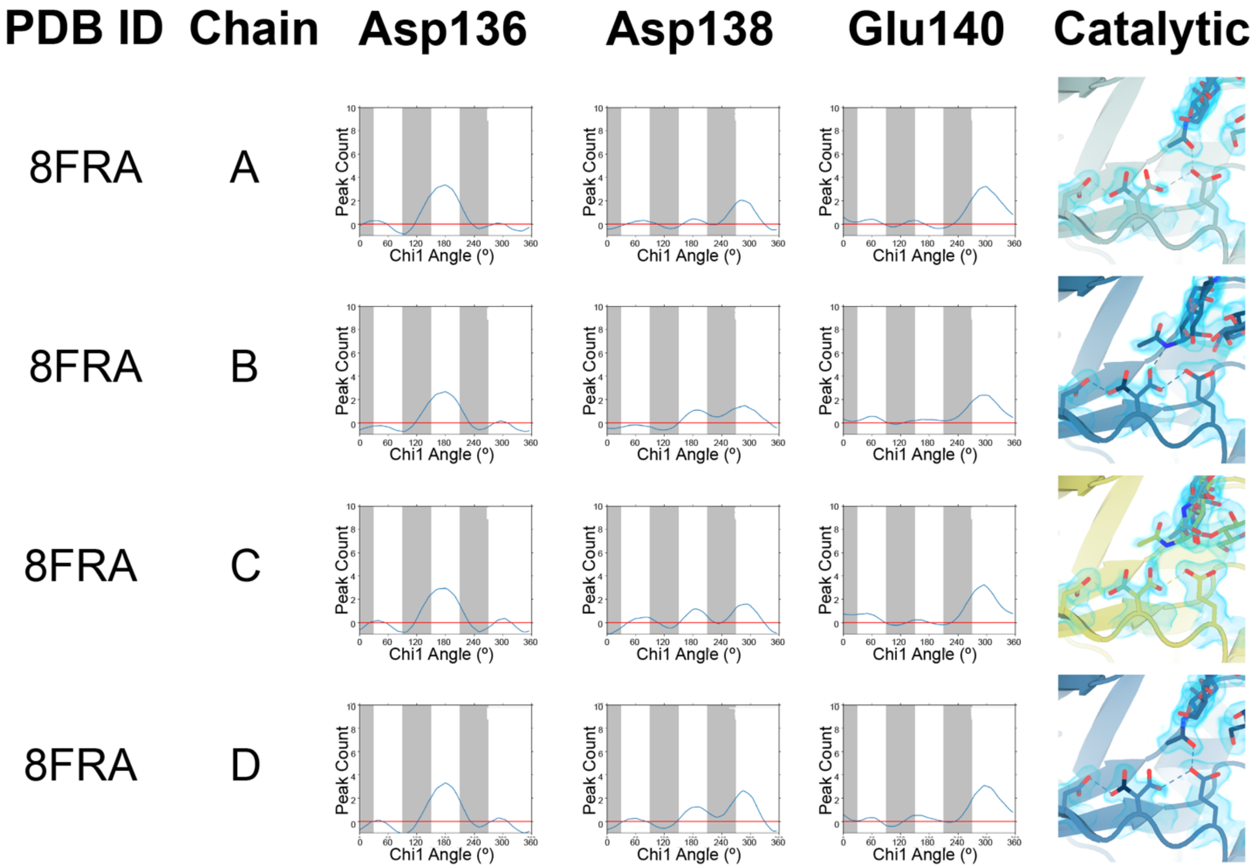

Supplemental Figure 7 | Ringer analysis of catalytic triad confirms alternative Asp138 conformations.

A) Ringer analysis to detect alternative conformations in electron density maps. Ringer detected one peak for Asp136 at χ_1 = 180º and Glu140 at χ_1 = 300º, indicating only one conformation, whereas two peaks were detected for Asp138 at χ_1 = 180º and χ_1 = 300º, indicating two alternative conformations. **B)** Stick representation of Asp136, Asp138, and Glu140 with 2mFo-DFc map volume shown as a 1.2 Å contour (blue).
